# Comprehensive analysis of amino acid sequence diversity at the F protein cleavage site of Newcastle disease virus in fusogenic activity

**DOI:** 10.1371/journal.pone.0183923

**Published:** 2017-09-01

**Authors:** Yanhong Wang, Wanqi Yu, Na Huo, Wenbin Wang, Yuanyuan Guo, Qiaolin Wei, Xinglong Wang, Shuxia Zhang, Zengqi Yang, Sa Xiao

**Affiliations:** College of Veterinary Medicine, Northwest A&F University, Yangling, Shaanxi, P.R. China; University of Maryland at College Park, UNITED STATES

## Abstract

Newcastle disease virus (NDV) is a contagious agent of Newcastle disease in avian species and seriously affects the poultry industry. The cleavage site of the viral F protein (Fcs) is a key determinant of membrane fusion and viral virulence. In this study, we investigated the precise effect of variable amino acid sequences of the Fcs on fusogenic activity. Based on viral pathogenicity, the Fcs sequences of natural isolates (n = 1572) are classified into eight types of virulent Fcs (VFcs) with the motif “G/R/K-R-Q/R/K-R/K-R↓F” and ten types of the avirulent Fcs (AFcs) with the motif “G/R/E-R/K/Q-Q-G/E-R↓L”. The VFcs is only found in the Class II cluster of viral classification and not in Class I. The AFcs exists in both Class I and II isolates. The VFc and AFc types present an evolutionary relationship with temporal distribution and host species. Using a fusion assay *in vitro*, VFcs-1 “RRQKR↓F” and VFcs-2 “RRQRR↓F” show the highest efficiency in triggering membrane fusion. The neutral residue Q at the P3 position of the VFcs plays an enhancing role compared to effect of the basic residues R and K. A single residue K at P3 or P5 is less efficient of the fusogenic activity in the VFcs with all basic residues. Moreover, the cleavage efficiencies of F_0_ proteins with different types of Fcs motifs do not appear to affect membrane fusion. Our findings offer insight into the effect of amino acid variation of the Fcs on the fusion triggered by NDV.

## Introduction

Newcastle disease (ND) is one of the most important infectious diseases in avian species and causes significant economic losses in the poultry industry worldwide [1]. Its causative agent is Newcastle disease virus (NDV), also called avian paramyxovirus serotype 1 (APMV-1), a member of the genus *Avulavirus* of the family *Paramyxoviridae*, encompassing a diverse group of single-stranded, negative-sense RNA genomes [2]. The genome consists of six genes in the order 3’-NP-P-M-F-HN-L-5’, which encode six proteins, namely, nucleocapsid (N) protein, phosphoprotein (P), matrix (M) protein, fusion (F) protein, hemagglutinin-neuraminidase (HN) and large polymerase protein (L). The F protein is a transmembrane glycoprotein responsible for viral envelope fusion with host cell membranes, allowing viral entry into the cytoplasm of target cells to cause viral spread and cell-cell membrane fusion [3]. The HN protein is a type II homotetrameric glycoprotein responsible for virus attachment to host cell receptors, which are ubiquitous sialic acid-containing macromolecules [4]. In NDV, the F protein alone cannot activate membrane fusion; instead, fusion is accomplished by HN participation [5].

Although NDV virulence is determined by multiple genetic factors, the F protein cleavage site (Fcs) is known as a major determinant [6]. The activation of the F protein is the key for virus entry into host cells and fusion with adjacent cells, resulting in giant, multinucleated cells or syncytia [7]. The paramyxovirus F protein is synthesized as the inactive precursor F_0_, which must be proteolytically cleaved by cellular proteases to produce two disulfide-linked subunits, F_1_ and F_2_, which are required for membrane fusion [8]. The Fcs of virulent NDV strains has a multi-basic amino acid cleavage motif, “^112^(R/K)-R-Q-(R/K)-R↓F^117^” (R, arginine; K, lysine; Q, glutamine; G, glycine; F, phenylalanine; Arrow, cleavage position; Number, residue position in F protein), and is a preferred recognition site for the host protease furin “R-X-(R/K)-R↓” (X, any residue). The furin is a ubiquitous intracellular protease in most cell types that results in systemic infection of virulent virus [9]. The Fcs sequences of avirulent strains have a monobasic amino acid cleavage motif, “^112^(G/E)-(R/K)-Q-(G/E)-R↓L^117^”, that requires extracellular secreted proteases, such as trypsin-like proteases, for cleavage and formation of syncytia in cultured cells. Therefore, avirulent virus causes subclinical infection in respiratory and enteric tract diseases [10, 11].

Amino acid variation in Fcs can dramatically affect membrane fusion. The dibasic structure, especially the R residues at positions P4 and P1, the K or R residue at P2 and the F residue at P1’, are necessary for the host-cell protease to cleave F_0_ and to form syncytium after co-transfection of cultured cells with F and HN genes [12, 13]. Modification of Fcs can significantly alter the fusion and pathogenicity of recombinant NDV. Avirulent virus with Fcs “GRQGR↓L” replaced by the virulent strain Fcs “RRQRR↓F” increased viral pathogenicity [6]. In contrast, in a virulent strain with the Fcs “RRQRR↓F” with G residues substituted at positions P5, P4 and P2, and L at the P1’ position, none of the Fcs mutants formed syncytium and cytopathic effect (CPE) in avian cells [10]. The virulent virus with the Fcs of avirulent virus was unable to cause membrane fusion and virulence [14]. Moreover, recombinant viruses with alteration of the Q residue at position P3 of the Fcs had reduced viral pathogenicity [15]. Furthermore, the APMV-2 virus with the Fcs “KPASR↓F” that was mutated to all basic R residues of “RRRRR↓F” produced syncytia sizes larger than the mutant Fcs “KRKKR↓F”. These results indicated that the R residue is preferred over K for cleavage by a host cell protease; the number but also the type of basic residue influences the cleavage of F0 protein [16].

Although the amino acids of the Fcs affect membrane fusion, the precise role of a variable Fcs is not well understood. In this study, we investigated the diversity of amino acid sequences at the Fcs motifs of NDV natural isolates (n = 1572). The Fcs motifs were classified into eight types of VFcs and ten types of AFcs based on the pathogenicity of the isolates. The Fcs types show the relationship to temporal distribution and avian species. Using F genes from virulent and avirulent strains as backbones in transfected susceptible cells, the neutral residue Q at position P3 in the VFcs increased the efficiency of membrane fusion compared to that of the basic residues R and K. The single basic residue K at position P3 or P5 was less efficient at fusogenic activity in the VFcs with all basic residues. Our data provide a comprehensive analysis of the Fcs and the efficacy of individual amino acids in fusion.

## Materials and methods

### Sequence analysis of the F protein

A total of 1,590 F gene sequences of NDV natural isolates were collected from the GenBank database and previous reports [17, 18]. The F protein information is summarized in Supplementary S1 Table. Sequence logos of amino acids at the F protein cleavage site were analyzed to display the position-specific features of multiple sequence alignments [19], which were generated using Weblogo3.1 (http://weblogo.threeplusone.com).

### Cells and virus

BHK-21 cells (ATCC, USA) were grown in Dulbecco’s modified Eagle medium (DMEM) supplemented with 10% heat-inactivated fetal bovine serum (FBS; HyClone) and were maintained in DMEM with 5% FBS, 100 U of penicillin/ml and 10 μg of streptomycin/ml at 37°C in a 5% CO_2_ incubator. NDV strain F48E9 is a virulent strain that is widely used as a standard challenge virus in China. The LaSota strain is an avirulent vaccine virus. The virus was propagated in 9-day-old specific pathogen-free (SPF) embryonated chicken eggs.

### Plasmids expressing F and HN

Viral RNA of the strains F48E9 and LaSota was extracted using TRIzol reagent according to the manufacturer’s instructions. The F or HN genes of NDV strains were amplified by reverse transcription-PCR (RT-PCR) with specific primers and four-pair appropriate primers (5’-3’), F48E9-FF (CGAGCTCATGGGCCCCAAATCTT), and FR (CGCTCGAGTCACATTCTTGTAGTGGC), F48E9-HNF (CGAGCTCATGGACCGTGTAGTTAG), and HNR (CGCTCGAGTTAAATCCCATCATCCT). LaSota-FF (CCGCTCGAGACCATGGGCTCCAGACCTTCTACCAAGAACC), and FR (GAAGATCTTCACATTTTTGTAGTGGCTCTCATCTGATCTAG), LaSato-HNF (CCGAGCTCACCATGGACCGGCCCGTTAGCCAAGTTGC), and HNR (GAAGATCTCTAGCCAGACCTGGCTTCTCTAACC), which were based on published sequences in GenBank (Accession No. AY508514 for F and AY034892 for HN of the F48E9 strain, DQ195265 for F and FJ004152 for HN of the LaSota strain). The amplified F and HN genes were cloned into the eukaryotic expression vector pCAGGS (pCAG) using restriction sites SacI and Xho I as pCAG-F and pCAG-HN for the F48E9 strain and restriction sites Bgl II and Xho I and Sac I and Bgl II as pCAG-F and pCAG-HN for the LaSota strain. The cloned genes were sequenced by GENEWIZ Inc., China. The VFcs and AFcs mutants were generated using overlap PCR. All F mutants were inserted into pCAGGS with the addition of a Flag tag (DYKDDDDK) at the C terminus of the F protein for detection.

### Transfections

BHK-21 cells were plated at 2×10^5^ per well in a 24-well plate for a 24-h incubation. The cells were co-transfected with equal amounts of pCAG-F or Fcs mutants and pCAG-HN using TurboFect Transfection Reagent (Thermo Fisher). Briefly, 0.8 μg of plasmids and 1.6 μl of transfection reagent in 0.1 ml of Opti-MEM (Gibco) was mixed for 15–20 min at room temperature; the DNA complex was added dropwise to the plate and incubated for 48 h.

### Fusion assay

The fusogenic capacity of the mutant F protein was determined by evaluating syncytium formation. BHK-21 cells in 24-well plates were co-transfected with 0.4 μg of pCAG-F or F mutants and 0.4 μg of pCAG-HN. Forty-eight hours after transfection, the numbers of nuclei in 40 fusion areas were counted to determine the average syncytia size, as previously described [20]. The average syncytia size was calculated as the ratio of the total number of nuclei in multinuclear cells to the total number of nuclei in the field [12, 21]. Data shown are averages of three independent experiments. Error bars refer to standard errors of the means (P<0.01, reflects the comparison of all groups through ANOVA).

### Western blot

The transfected BHK-21 cells were lysed in RIPA buffer with the protease inhibitor PMSF on ice for 10 min. The cell lysates were subjected to 10% SDS-PAGE and transferred to a PVDF membrane (Millipore, USA). Anti-Flag monoclonal antibody (CoWin Biosciences, China) at 1:3000 dilution was used as a primary antibody and was incubated with the membrane. The secondary antibody was Goat anti-mouse IgG conjugated with horseradish peroxidase (Sungene Biotech, China). The ECL peroxidase substrate (Millipore) was used for detection.

## Results

### Diversity and classification of amino acid sequences of F cleavage sites

The 1590 F gene sequences of NDV natural isolates were collected from the GenBank database and previous reports. Some isolates lacking pathogenicity information and some possessing unconventional amino acid sequences in the Fcs were deleted. Therefore, a total of 1572 isolates were used for Fcs analysis. Based on the pathogenicity of these natural isolates, the Fcs was classified into eight types of virulent Fcs (VFcs) with 1073 isolates in Table 1 and ten types of avirulent Fcs (AFcs) with 499 isolates in Table 2. All eight types of VFcs belonged to Class II NDV. The VFcs-1 “RRQKRF” (801 isolates) was the most dominant type of VFc and covered most genotypes of Class II except for genotypes I, III, VI and XV. The VFcs-4 “GRQKRF” (5 isolates) and VFcs-7 “KRKKRF” with one isolate belonged only to genotype VI. The VFcs-3 “KRQKRF” and -5 “RRKKRF” presented in genotypes V, VI and VII only existed in virulent isolates. The VFcs-8 “RRRRRF” was specific to the genotype XI isolates. All VFcs have an F residue at P1’. On the basis of the amino acid characteristics at P1 to P5, the eight types of VFcs were divided into two groups: Group 1 with polybasic residues (VFcs-1 to -4) and Group 2 with all basic residues (VFcs-5 to -8). Importantly, the Q residue at P3 was unique in Group 1.

**Table 1:** Cleavage sites of virulent NDV F proteins from natural isolates.

Group	Category	F cleavage site^*a*^ (P_5_P_4_P_3_P_2_P_1_↓P _1_’)	The number of strains	Genotype^*b*^
	VFcs-1	RRQKR**↓**F	801	II, V-VIII, XII-XIV, XVI-XVIII
**1**	VFcs-2	RRQRR**↓**F	56	I, III-IV, VII, IX, XIII, XVII
	VFcs-3	KRQKR**↓**F	51	V-VII
	VFcs-4	GRQKR**↓**F	5	VI
	VFcs-5	RRKKR**↓**F	86	VI-VII
**2**	VFcs-6	RRRKR**↓**F	59	VI-VII, XIII-XIV, XVII-XVIII
	VFcs-7	KRKKR**↓**F	1	VI
	VFcs-8	RRRRR**↓**F	14	XI

^***a***^ A virulent virus is defined as a virus with a polybasic amino acid sequence and a phenylalanine (F) residue at P1’ at the Fcs. P5 to P1’ represent the position (P) of each residue. Arrow indicates cleavage by protease between P1 and P1’.

^***b***^ Roman numerals represent Class II genotypes.

**Table 2:** Cleavage sites of avirulent NDV F proteins from natural isolates.

Category	F cleavage site (P_5_P_4_P_3_P_2_P_1_↓P _1_’)	The number of strains	Genotype^*b*^
AFcs-1	GRQGR**↓**L	143	Class I, I-II
AFcs-2	GKQGR**↓**L	121	I-II, X
AFcs-3	RRQGR**↓**L	3	I
AFcs-4	ERQER**↓**L	156	Class I
AFcs-5	RRQGR**↓**F^*a*^	1	I
AFcs-6	ERQGR**↓**L	5	Class I, I
AFcs-7	RKQGR**↓**L	2	I
AFcs-8	EKQGR**↓**L	22	I, X
AFcs-9	EQQER**↓**L	44	Class I
AFcs-10	RRQRR**↓**L	2	I

^***a***^ These isolates have avirulent phenotypes in birds, consistent with the deduced avirulent Fcs [22].

^***b***^ No Class I genotype is included. Roman numerals represent Class II genotypes.

All ten types of AFcs possessed mono- or multi-basic amino acids at P1 to P5 and an L residue at P1’, except for AFcs-5, which has an F residue in Table 2. These AFcs motifs all belonged to Class I and Class II NDV. The AFcs-1 “GRQGRL”, -2 “GKQGRL” and -4 “ERQERL” were the most prevalent AFcs, with 143, 121 and 156 isolates, respectively. However, the AFcs-2 only existed in Class II, and AFcs-4 was only found in Class I. The AFcs-8 “EKQGRL” belonged to genotypes I and X of Class II, and AFcs-9 “EQQERL” only existed in Class I. Interestingly, the AFcs-3, -5, -7 and -10, present in fewer isolates, only existed in genotype I of Class II. These findings denoted more varied genotypes in the VFcs than in the AFcs. Notably, the AFcs-5, an Australian isolate with the motif “RRQGRF”, was expected to be a VFcs based on an intracerebral pathogenicity index (ICPI) of 1.68 and polybasic residues with a single F residue at P1’. However, this isolate was defined as an avirulent virus because the inoculated birds presented the avirulent phenotype [22]. Taken together, these data indicate that the Fcs of NDV present a diversity of amino acid sequences and enable classification into eight types of VFcs and ten types of AFcs.

### Temporal and geographic distribution of F cleavage site types

**Fig 1:**
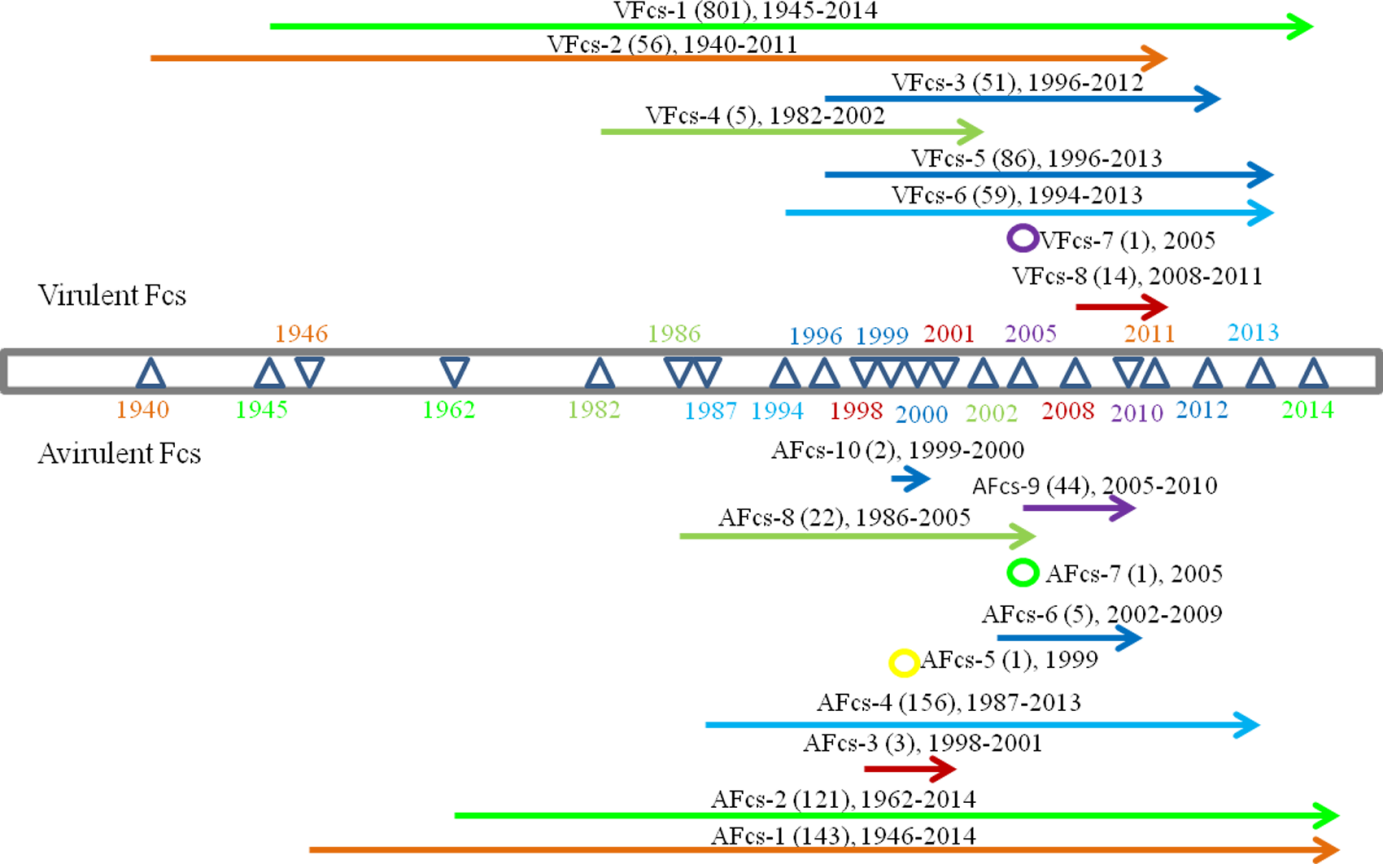
Timeline of Fcs variants in NDV natural isolates. F cleavage sites are illustrated in Tables 1 and 2. Each triangle represents a year of time. The upper panel indicates information for virulent Fcs, and the lower panel indicates avirulent Fcs.

Since the first discovery of NDV in 1926, numerous strains have been naturally isolated worldwide. To determine the epidemiological trends in Fcs types, the eight types of VFcs and ten types of AFcs were analyzed using a timeline (Fig 1). In the VFcs, VFcs-1 and VFcs-2 include 801 and 56 isolates, respectively, and have been the most distributed types for approximately 70 years (since the 1940s) on most continents. VFcs-3, VFcs-5 and VFcs-6 appeared from the 1990s to the present. VFcs-8 was sporadically distributed only in Madagascar from 2008 to 2011. VFcs-7 consisted of only a single isolate found in the USA in 2005. VFcs-4 existed for 20 years in Italy, Great Britain, China, Argentina and the USA but was not found after 2002. Among the AFcs, AFcs-1, AFcs-2 and AFcs-4 have been widely observed from the 1940s to the present. AFcs-6 and AFcs-9 were mainly isolated from China and the USA from 2002 to 2010. AFcs-3 was sporadically present in Australia. In 1999 and 2005, single AFcs-5 and AFcs-7 strains were isolated in Australia and the USA, respectively. These data suggested that although the VFcs and AFcs of NDV isolates could undergo the co-evolution, recent prevalent Fcs types were presented only in individual geographic areas.

### Variation of F cleavage site in avian species

**Fig 2:**
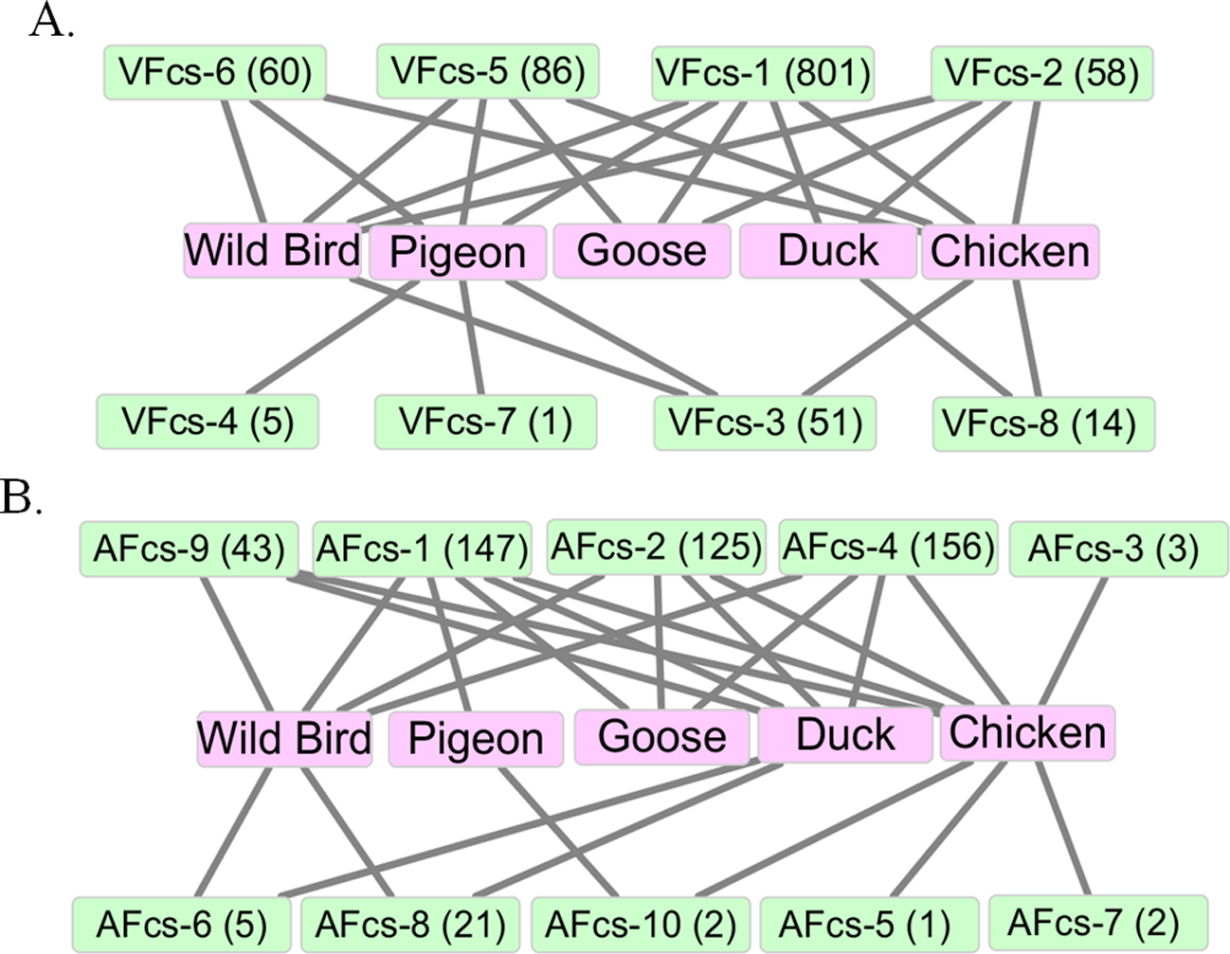
Relationship between Fcs variants and avian species. Birds except chicken, pigeon, goose and duck are considered wild birds. A. Virulent Fcs in avian species. B. Avirulent Fcs in avian species.

NDV strains have been mostly isolated from avian species, including domestic and wild birds. The species-specific original NDV isolates showed varied virulence or pathogenicity across avian species. To assess whether the Fcs as a virulent factor relates to host species, the birds and Fcs types were statistically described in Fig 2. Chickens and wild birds were the most common species for isolated NDV strains, as they contained five and six types of VFcs, respectively (Fig 2A). VFcs-1, VFcs-2 and VFcs-5 were found in isolates from those four species and wild birds. The duck- and goose-origin isolates contained common VFcs-1 and VFcs-2 and specific VFcs-8 and VFcs-5, respectively. Notably, VFcs-4 and VFcs-7 only existed in pigeon-origin isolates. Interestingly, the pigeon-origin isolates had VFcs-1, VFcs-3, VFcs-4, VFcs-5, VFcs-6 and VFcs-7, which contained a common K residue at P2.

In the AFcs, AFcs-1, AFcs-2 and AFcs-4 existed in the largest number of isolates and involved almost all host species (Fig 2B). Although the isolates from pigeons only contained AFcs-1 and AFcs-10, most pigeon-origin isolates were virulent. Meanwhile, from the types AFcs-1 to AFcs-10, there all contained the duck-origin isolates. These results indicated that most AFcs were specifically related to waterfowls.

### Variation of amino acids in the F cleavage site

**Fig 3:**
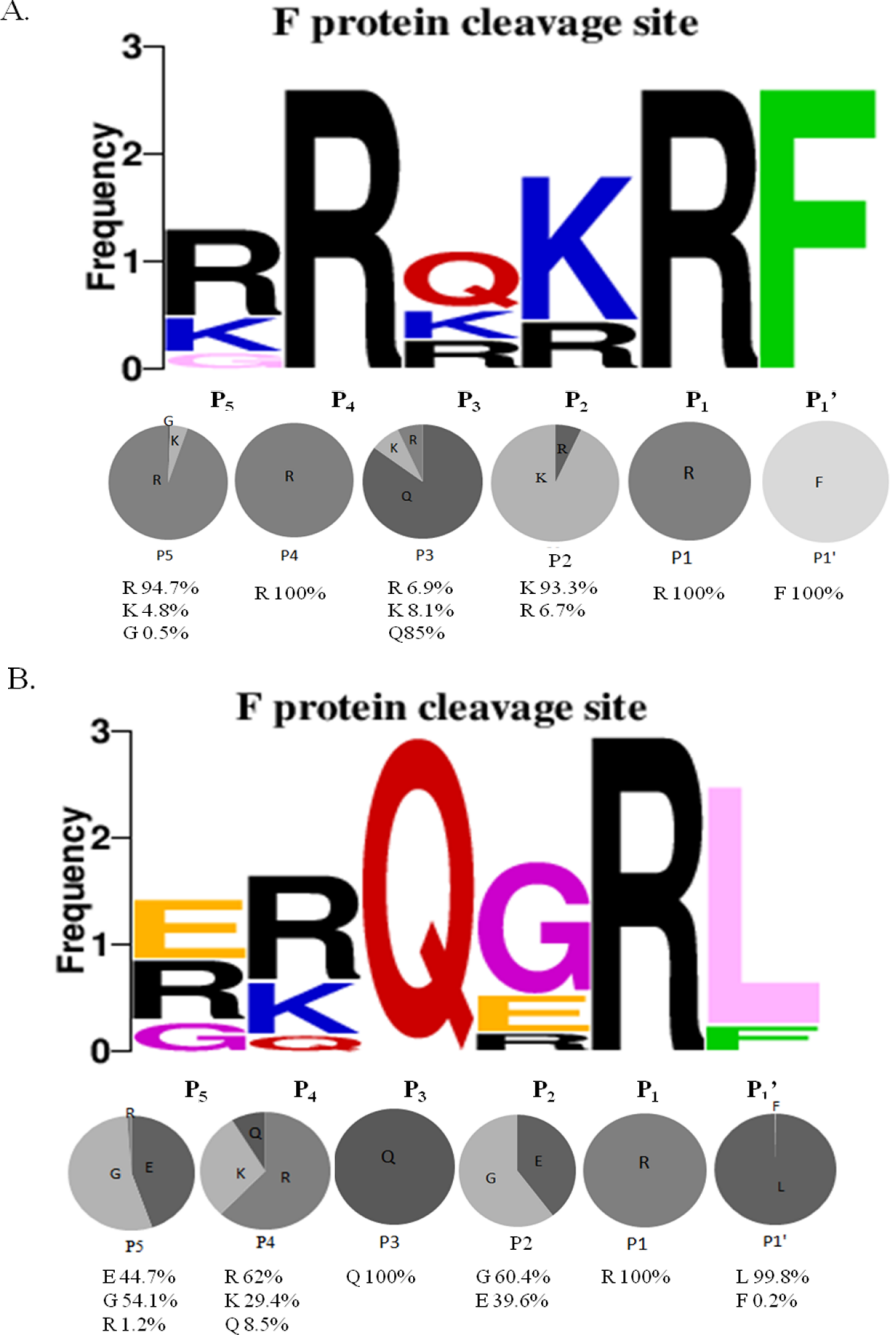
Sequence analysis of Fcs variants at the fusion protein cleavage site in Newcastle disease virus (NDV). Diversity of residues at each position of the Fcs were analyzed using WebLogo 3.1 (http://weblogo.threeplusone.com/create.cgi). (A) Fcs sequences from 1,073 virulent isolates. (B) Fcs sequences from 499 avirulent isolates. Upper panel: frequency of each residue at a given position. Lower panel: Percentages of each amino acid represented in pie graphs.

Based on the F protein sequence analysis, a total of 1,073 natural isolates possessed the virulent cleavage site motif “^112^G/R/K-R-Q/R/K-R/K-R-F^117^” (Fig 3A), which contained the sequence motif “R-X-R/K-R”, which is recognized by furin protease. The basic R residues at P1 and P4 and the F residue at P1’ were extremely conserved, with frequencies of 100% in all VFcs. The K and R residues at P2 were present at frequencies of 93.3 and 6.7%, respectively. At the P3 position, the Q residue was the most frequent at 85%, but the K and R residues were less frequent at 8.1% and 6.9%, respectively. At the P5 position, the R residue was the most frequent at 94.7%, but the K (4.8%) and G (0.5%) residues were less frequent in the VFcs. These data revealed that the highly conserved R residue at P4 and the F residue at P1’ are important for structural recognition of the VFcs by host furin.

A total of 499 natural isolates possessed the avirulent cleavage site motif “^112^G/R/E-R/K/Q-Q–G/E-R-L^117^”, which was unable to be recognized by furin (Fig 3B). The residue Q at P3 and the R residue at P1 were extremely conserved, with frequencies of 100%. A clear majority of the hydrophobic amino acid residue L (99.8%) at P1’ was mostly conserved. However, the G (60.4%) at P2, R (62%) at P4 and G (54.1%) at P5 were not present at high frequencies, and the Q (8.5%) at P4 and R (1.2%) at P5 were less frequent. These data indicated that the residues at P2, P4 and P5 were variable, but the Q residue at P3 and the L residue at P1’ were critical for maintaining the characteristics of the AFcs, which could not be cleaved by furin.

### Cell membrane fusion induced by both F and HN in a dose-dependent manner

**Fig 4:**
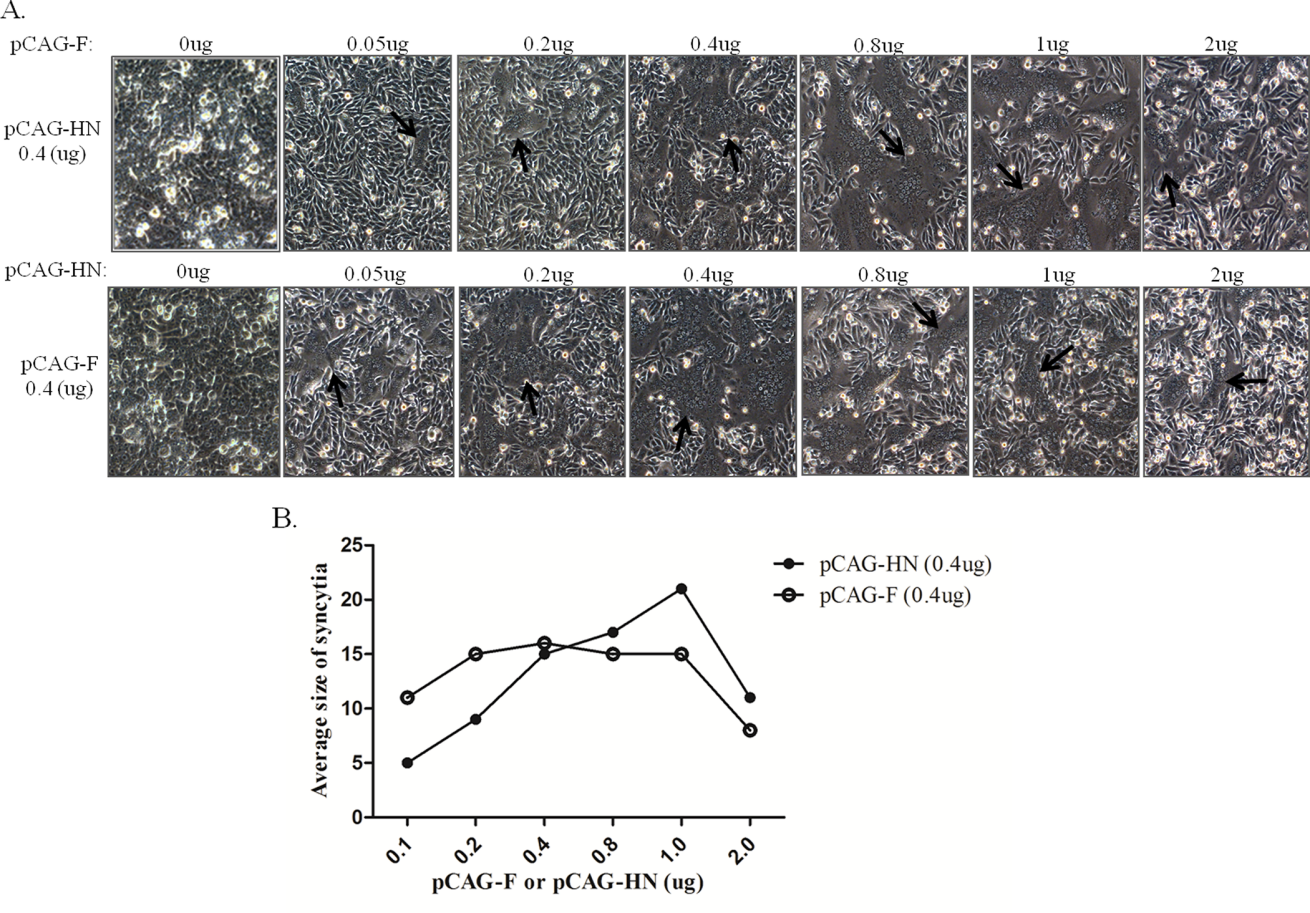
Dose-dependent induction of syncytia formation by F and HN proteins in BHK-21 cells. Cells were co-transfected with different amounts of pCAG-F and pCAG-HN. Syncytia were counted based on nuclei. (A) Syncytia formation is indicated by black arrows. (B) Syncytia were quantitated using the average size method.

Both F and HN proteins of NDV are involved in cell-cell membrane fusion. To assess the amounts of F and HN proteins involved in triggering fusion activity, BHK-21 cells were co-transfected with different amounts of two plasmids containing F and HN from the virulent F48E9 strain. With constant addition of 0.4 μg of pCAG-HN, the syncytia were smaller with the addition of pCAG-F at 0.05 and 0.2 μg. Larger syncytia were formed with 0.4, 0.8 and 1 μg of pCAG-F but decreased with the addition of 2 μg of pCAG-F (Fig 4A). In contrast, syncytia size was similarly induced with the addition of pCAG-HN at 0.2, 0.4, 0.8 and 1 μg with the constant addition of 0.4 μg of pCAG-F but decreased with the addition of pCAG-HN at 0.05 and 2 μg (Fig 4B). These results indicated that syncytia formation was dependent on the amounts of F and HN, and the induction of syncytia was more efficiently interfered by F than by HN protein.

### Effect of a virulent F cleavage site on cell membrane fusion

**Fig 5:**
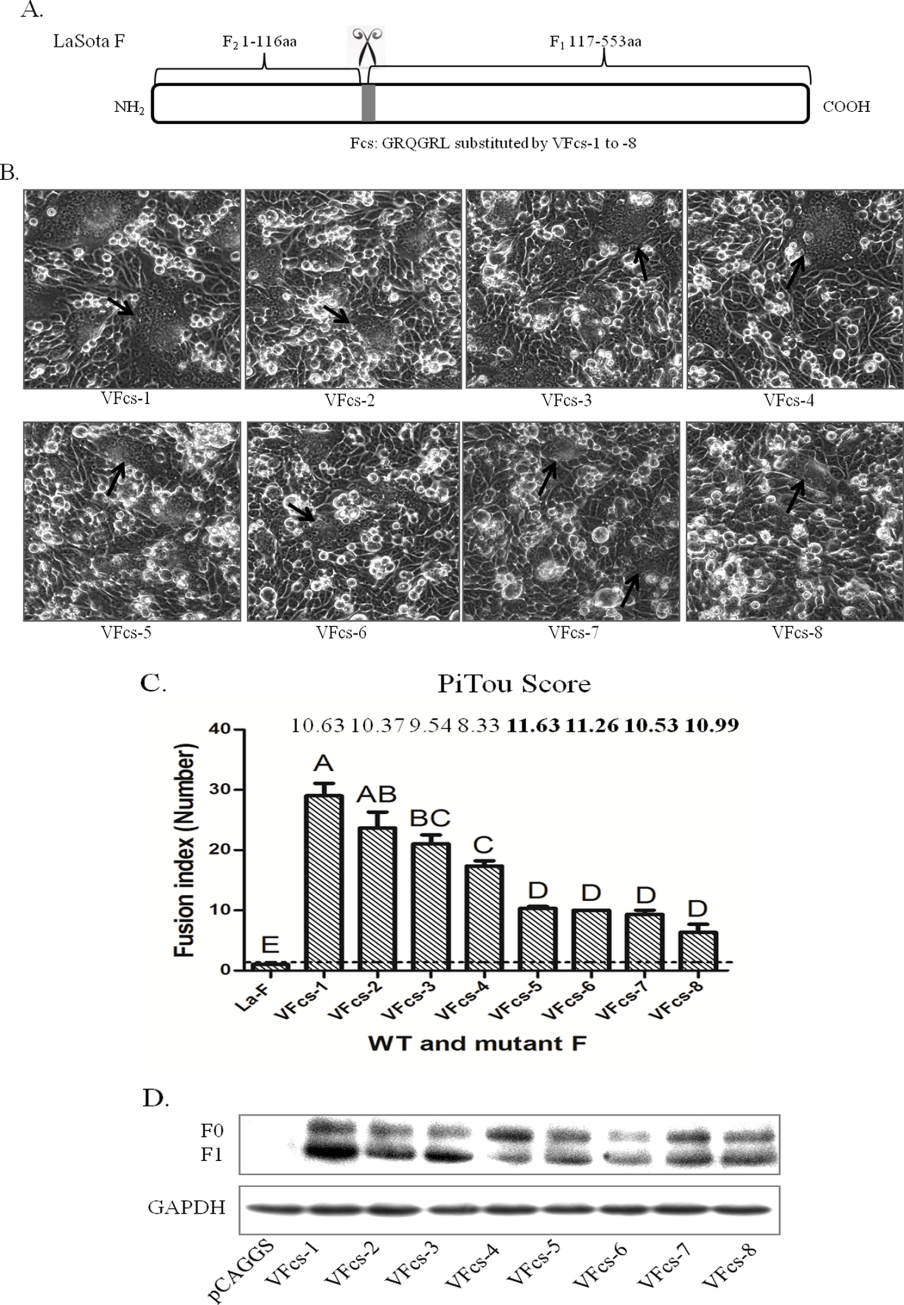
Syncytium formation induced by virulent Fcs and HN in BHK-21 cells. (A) Schematic of avirulent Fcs substituted with VFcs-1 to VFcs-8 in the LaSota F backbone. (B) Syncytium formation induced by co-transfection with pCAG-Fcs mutants and pCAG-HN of LaSota (0.4 μg of F and 0.4 μg of HN). Syncytia are indicated by black arrows. (C) Quantitation of syncytium formation. BHK-21 cells were co-transfected with pCAG-Fcs mutants and pCAG-HN using Turbofect as described in the Materials and Methods. The number of nuclei in 40 fusion areas was counted at 48 h post-transfection to determine the average syncytia size. Data are the average of syncytia numbers in three independent experiments where statistical significance is designated with capital letters and different letters indicate significant differences (P<0.01). Furin prediction scores of cleavage in Fcs variants were analyzed by Pitou (http://www.nuolan.net/reference.html). (D) Proteolytic cleavage of the F protein with Fcs variants. BHK-21 cells were co-transfected with 0.4 μg of pCAG-F with different Fcs and 0.4 μg of pCAG-HN. The F protein was analyzed via Western blot at 48 h post-transfection. GAPDH protein expression is shown as a control.

Generally, virulent strain F with HN participation can induce cell-cell membrane fusion, but not in the avirulent strain. This effect is due to the fact that the virulent strain F protein has polybasic amino acids in the Fcs, which can be recognized and cleaved by host proteases such as furin. In contrast, the avirulent strain Fcs is unable to be cleaved by furin. To evaluate the effects of VFcs-1 to VFcs-8 on cell membrane fusion, the F gene of the avirulent LaSota strain was used as a backbone to construct VFcs mutants that can easily determine membrane fusion activity. The Fcs “GRQGRL” of the LaSota strain was respectively substituted by VFcs-1 to VFcs-8 (Fig 5A). Apart from the wild type LaSota-F protein, syncytium formation was observed by co-transfection with Fcs mutants and HN of the LaSota strain in accordance with the ratio of 1:1 (Fig 5B). The VFcs-1 to VFcs-4 mutants with Q residues at P3 showed larger syncytia than VFcs-5 to VFcs-8 mutants (P<0.01). The syncytia induced by VFcs-1 and VFcs-2 mutants with R at P5 were more efficient than VFcs-4 mutants (Fig 5C). Meanwhile, there was no significant difference in all basic residues (P1 to P5) of VFcs-5 to VFcs-8, and the syncytia were significant difference compared with the VFcs-1 to VFcs-4 mutants. Thus, the basic residue R or K at P3 in VFcs significantly affected syncytium formation. The peptide cleavage scores generated by a Pitou 2.0 furin cleavage prediction algorithm were used to represent the solvent accessibility and the binding strength of protein sequence residues, with a higher score predictive of a stronger degree of cleavage [23]. The trends of VFcs-1 to VFcs-4 cleavage scores were consistent with an experimental fusion assay. However, the PiTou data were inconsistent with fusion index among Group 2 (VFcs-5 to VFcs-8) (Fig 5C). The predictive precision of the Pitou score is unknown in this case. These results demonstrated that Group 1, with a Q residue at P3, was more efficient in cell membrane fusion activity than Group 2.

To examine whether different types of VFcs affect F_0_ protein cleavage, BHK-21 cells were co-transfected with F_0_ containing VFcs and the HN of the LaSota strain. The Western blot showed that the F protein with the VFcs motifs were partially cleaved into F_1_ by host proteases, but no significant difference in F_1_/F_0_ ratios was found between the eight VFcs (Fig 5D). These results implied that the cleavage efficiency of the F_0_ protein was not affected by variation in the VFcs in the cells.

### Lysine (K) at P3 in the VFcs of Group 2 is critical for induction efficacy of cell membrane fusion

**Fig 6:**
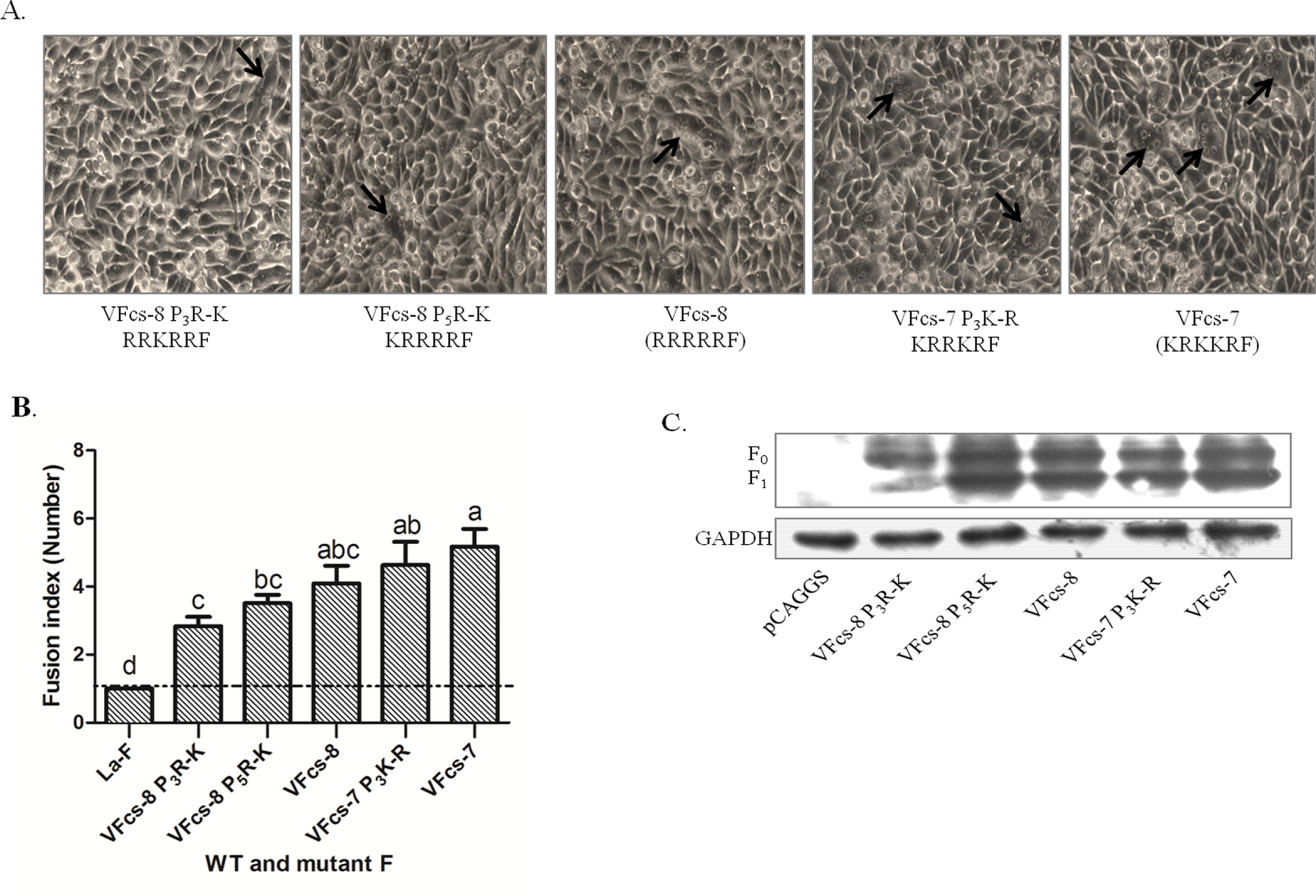
Membrane fusion capacity induced by virulent Fcs with all basic residues. (A) VFcs-7(P3_K-R_), VFcs-8(P3_R-K_) and VFcs-8(P5_R-K_) were transfected into DF1 cells with wild-type HN. Syncytia are indicated by black arrows. (B) Average syncytia size was determined. Results are the average of three different experiments where statistical significance is designated with lowercase letters and different letters indicate significant differences (P<0.05). (C) Proteolytic cleavage of F protein mutants co-transfected with wild-type HN in BHK-21 cells, as analyzed via Western blot. GAPDH protein expression is shown as a control. Proteins were detected by Western analysis with anti-Flag antibody. F0, uncleaved fusion protein; F1, cleaved F0 product.

In Group 2, VFcs-5 to VFcs-8 consist of all basic residues (R/K) at P1 to P5 of the VFcs. To further analyze the effects of R and K residues in the VFcs with all basic residues on cell membrane fusion activity, the R residues at P3 and P5 of VFcs-8 were artificially mutated to K residues, and the resulting constructs were named VFcs-8 P3_R-K_ and VFcs-8 P5_R-K_, respectively. The K at P3 of VFcs-7 was mutated to an R residue and was named VFcs-7 P3_K-R_. BHK-21 cells were co-transfected with the VFcs mutants and HN of the LaSota strain. The syncytia induced by these VFcs mutants were small and few (Fig 6A). The average syncytia size was slightly decreased in the VFcs-8 P3_R-K_ and VFcs-8 P5_R-K_ mutants compared with that in VFcs-8. The VFcs-7 and VFcs-7 P3_K-R_ mutants showed slight differences in syncytium formation. However, the fusion activity induced by VFcs-7 was significantly higher than that of VFcs-8 P3_R-K_ and VFcs-8 P5_R-K_ (P<0.05) (Fig 6B). These results suggested that VFcs with a single residue K at P3 or P5 was less efficient to induce the membrane fusion than other VFcs mutants. This result was confirmed by Western-blotting assay, in which F_0_ protein cleavage of the VFcs-8 P3_R-K_ mutant was less efficient (Fig 6C).

### Effect of an avirulent F cleavage site on cell membrane fusion

**Fig 7:**
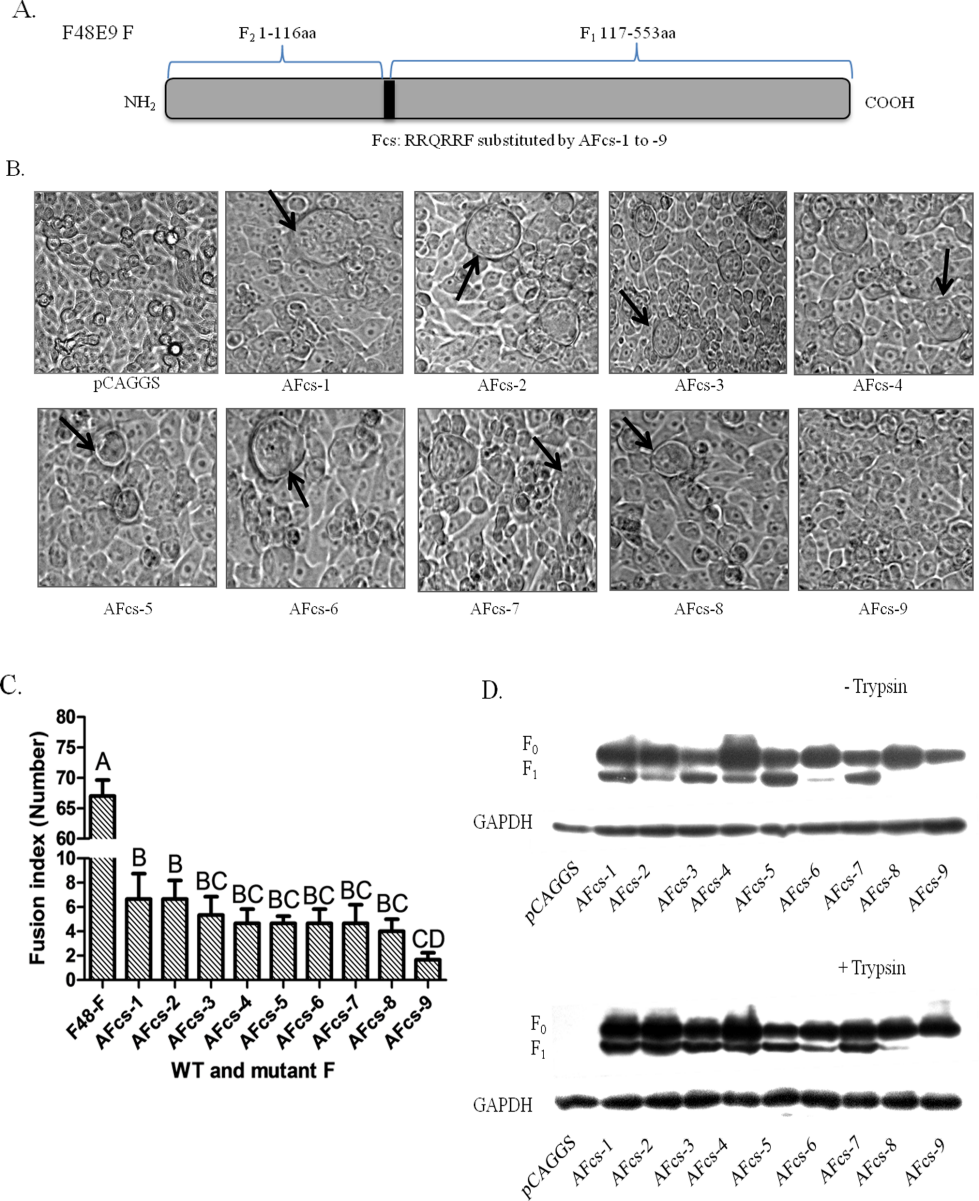
Syncytium formation induced by avirulent Fcs and HN in BHK-21 cells. (A) Schematic of virulent Fcs substituted with AFcs-1 to AFcs-9 in the F48E9 F backbone. (B) Syncytium formation induced by co-transfection with pCAG-Fcs mutants and pCAG-HN of F48E9 (0.4 μg of F and 0.4 μg of HN). Syncytia are indicated by black arrows. (C) Average syncytia size was determined in the presence of trypsin. Data are the average of syncytia numbers in three independent experiments where statistical significance is designated with capital letters and different letters indicate significant differences (P<0.01). (D) Proteolytic cleavage of the F protein with Fcs variants. BHK-21 cells were co-transfected with 0.4 μg of pCAG-F with different Fcs and 0.4 μg of pCAG-HN. The F protein was analyzed via Western blot at 48 h post-transfection. GAPDH protein expression is shown as a control.

The avirulent virus F protein with AFcs cannot induce cell membrane fusion. However, it can trigger membrane fusion with supplementation of exogenous proteases, such as trypsin. To analyze the effects of AFcs-1 to AFcs-10 on the induction of membrane fusion, the F gene of the virulent F48E9 strain was used as a backbone to construct AFcs mutants. Thus, the F48E9 strain Fcs “RRQRRF” was replaced with AFcs-1 to AFcs-10 (Fig 7A). To determine whether AFcs mutants could induce syncytium formation, BHK-21 cells were co-transfected with AFcs mutants and HN of the F48E9 strain in accordance with the ratio of 1:1. None of the AFcs mutants showed syncytium formation in the absence of exogenous trypsin. Markedly, all AFcs mutants showed syncytium formation in the presence of trypsin supplementation (Fig 7B), and the wild type F48E9 F protein showed the largest syncytia (65±2.5) compared with mutants. The AFcs-9 mutant was most weakly fusogenic, and the AFcs-1 and AFcs-2 mutants that were the most fusogenic and were significantly difference in syncytium formation than AFcs-9 mutant (P<0.01) (Fig 7C). This effect may be due to unique residues in AFcs-9 “EQQERL”, in which the Q residue at P4 was different from other AFcs motifs, which used an R or K residue. Suprisingly, the AFcs-10 “RRQRRL” mutant induced an extraordinarily high level of the syncytia at the average size of 28 (28±2.8) that was similar to the VFcs mutant induced syncytia. However, avirulent F with the AFcs-10 cleavage site did not induce or slightly induce the syncytia. This data suggested that other regions of the virulent F protein might contribute the membrane fusion. This phenomenon needs to be further investigated. The F_0_ cleavage of the AFcs mutants was analyzed in the absence and presence of trypsin via Western blot (Fig 7D). Although the F_0_ proteins of AFcs-1 to AFcs-7 were cleaved to F_1_ in the absence of trypsin supplementation (Fig 7D upper panel), syncytium formation was not observed. Cleavage of the AFcs-9 F protein was undetected in the presence of trypsin, just as in the absence of trypsin, possibly due to a low but undetectable level of F_1_ subunit, even though it had weakly fusogenic activity (Fig 7D lower panel). These results demonstrated that the F protein with AFcs cannot induce cell membrane fusion without exogenous protease and that the basic residues R or K at P4 are essential for efficient induction of fusogenic activity in the presence of exogenous protease.

## Discussion

The F protein of NDV co-participates with HN to trigger membrane fusion for viral entry into host cells. The cleavage site of the F protein as a key determinant is essential for fusion triggering. The types and properties of amino acids at the Fcs may have profound implications for fusogenic activity and proteolytic cleavage. Here, we analyzed natural isolates (n = 1572) with a diversity of amino acid sequences of the Fcs and classified them into eight types of VFcs and ten types of AFcs based on the pathogenicities of the isolates. Different types of VFcs and AFcs motifs existed in distinct temporal distributions and related to specific avian species. Using a fusion assay *in vitro*, the individual amino acids of the VFcs and AFcs were identified based on their contributions to fusion activity.

NDV isolates show varying clinical disease in chickens, from asymptomatic to high mortality. Therefore, they have been classified into velogenic and mesogenic (virulent) and lentogenic (avirulent) pathotypes based on their clinical signs and lesions. Fcs as virulence factors present different amino acid sequences in avirulent and virulent strains. In general, VFcs contain the motif “R/K-R-Q-R/K-R↓F”, and AFcs contain the motif “G/E-K/R-Q-G/E-R↓L”. The F and L residues at the P1’ position are clearly typical in VFcs and AFcs, respectively [24]. In our study, 1,073 virulent and 499 avirulent natural isolates were collected to extensively analyze the amino acid sequences of the Fcs. Our data revealed that the Fcs can be classified into eight types of VFcs with the motif “G/R/K-R-Q/R/K-R/K-R↓F” and ten types of AFcs with the motif “G/R/E-R/K/Q-Q-G/E-R↓L”. Within the VFcs, the Fcs was further divided into Group 1, which contains dibasic amino acids surrounding Q at P3, and Group 2, with five basic residues. The VFcs-1, VFcs-2 and VFcs-6 are the most dominant types of the VFcs that cover most genotypes of Class II virulent isolates. VFcs-4 and VFcs-7 in genotype VI were found, and VFcs-8 belongs to the genotype XI, indicating a specific linkage between Fcs type and genotypes. The AFcs types exist in Class I and genotypes I, II and X of Class II isolates, but none of the VFcs types exist in the Class I isolates, which explains why the Class I isolates are most avirulent. AFcs-1 and AFcs-2 have been the most distributed types from the 1940s to recent years, and other AFcs types have been found sporadically since the 1990s, suggesting that these Fcs types may evolve in geographic isolation for decades or result from relatively rapid evolution following vaccine escape.

NDV has a broad host range in domestic and wild birds that is attributable to native selection, host selective pressure, viral evolution or wide vaccination [17]. The Fcs may relate to the specific species from which the virus was isolated. Velogenic strains with VFcs were isolated from most avian species, except waterfowl such as duck, which are major species for the prevalently circulated lentogenic virus with AFcs. Notably, the VFcs of pigeon-origin velogenic or mesogenic isolates possessed a K residue at position P5, P3 or P2, perhaps due to natural ecology and the effect of selective pressure. Selection by the host usually led to an accumulation of additional beneficial mutations, resulting in the emergence of successful virus variants [25]. The VFcs on the virulent isolates may indicate successful viral adaptations to diverse poultry species.

Based on a fusion assay of the VFcs in the cells, Group 1, which contains dibasic amino acids, caused more syncytium formation than Group 2, with all basic residues, indicating that the Q residue at P3 is more important for increasing fusogenic activity than R or K residues. It is possible that the presence of a strong positively charged residue such as R or K at P3 of the Fcs may disturb the conformational stability of the furin binding site and influence cellular protease activity. Our results agreed with a previous report that a neutral residue Q at P3 maintained efficient proteolytic processes by host cell proteases [15]. In comparison to VFcs-1 to VFcs-4, replacement of the R residue with a G or K residue at P5 presented a marked decrease in cell-cell fusion. These results further confirmed that the R residues at P1, P2 and P4 were essential for cleavage of virulent F proteins, and for maximal cleavage to occur, an R residue at P5 and an F at P1’ were required, as previously described [13]. However, in the VFcs of Group 2, basic amino acids had no difference in syncytia size and cleavage of the F protein, inconsistent with a previous report, which showed different cleavage efficiencies for APMV-2 Fcs containing five R or K residues at positions P2, P3, and P5 [16], possibly due to different serotypes between NDV and APMV-2. In addition, all VFcs mutants showed analogous cleavage of the F_0_ protein, suggesting that cleavage efficiency of the F_0_ protein with different types of VFcs may not be significantly affected by the number and type of basic residues.

To evaluate the effect of the type of individual basic residue at P3 or P5 on fusion activity, an R residue artificially substituted by a K residue in the VFcs-8_P3R-K_ and VFcs-8 P5_R-K_ mutants have led less efficient fusion activity than in the VFcs-7. In the hypothetical two-dimensional model of furin substrate binding-site domains, the enzymatic subdomain of furin that interacts with the Q residue is not a distinct site. The substrate points away from the enzyme towards the solvent, whereas the enzymatic subdomains that interact with the basic residues of viral substrates are more distinct and form a defined pocket [26]. Our result may reflect that the K residue at the VFcs could influence the enzymatic subdomain of furin to form a pocket under the K residue at P3 or P5.

The F_0_ proteins of the AFcs mutants were cleavable in the absence or presence of trypsin supplementation in transfected BHK-21 cells. The AFcs mutants could induce syncytium formation in the presence of trypsin, but not in its absence. Similar results were found in a previous report: the recombinant virulent NDV Ban/AF strain with an AFcs “GRQGR↓L” did not produce syncytia, even though the F_0_ protein was cleaved in the presence or absence of exogenous protease. Recent studies have also shown that virulent NDV rMex mutant virus with an AFcs “GRQGRL” did not cause the syncytia in the absence or presence of added protease [14, 27]. It appears that the cleavage of the F_0_ protein is dispensable for cell-cell membrane fusion, suggesting that in addition to the cleavage site, other amino acid sequences in the F protein may contribute to fusion activity.

Finally, we found that the characteristics and positions of amino acids at the VFcs could directly increase fusion efficiency, especially a Q residue at P3. The single residue K at position P3 or P5 is less efficient of the fusion activity at VFcs with five basic residues. VFcs-1 and VFcs-2 are the most efficient for cell-cell membrane fusion activity, and the contribution of the VFcs to fusion efficacy occurs in the order VFcs-1 > VFcs-3 and VFcs-4; VFcs-2 > VFcs-4 > VFcs-5, VFcs-6, VFcs-7 and VFcs-8. The effects of these Fcs types on viral replication and pathogenicity will need to be explored further using recombinant virus with reverse genetics. Our findings offer insight into the precise role of Fcs in fusion triggering and contribute to the design of an effective protease inhibitor for antivirals.

## Supporting information

S1 Table The information of NDV F protein associated with this article. (XLSX)

## References

[1] Alexande DJ. Newcastle disease and other avian paramyxoviruses. A laboratory manual for the isolation and identification of avian pathogens American Association of Avian Pathologists, Kennett Square 1998;156–163.

[2] Mayo MA. Virus taxonomy–Houston. Arch Virol. 2002;147:1071–1076. doi: 10.1007/s007050200036 12021875 10.1007/s007050200036

[3] Lamb RA, Parks GD. Paramyxoviridae: the viruses and their replication In Knipe D. M. and Howley P. M. (ed.), Fields virology, 5th ed. Wolters Kluwer-Lippincott Williams & Wilkins, Philadelphia, PA 2007 pp.1449–1496.

[4] Nagai Y. Protease-dependent virus tropism and pathogenicity. Trends Microbiol. 1993;1:81–87. 8143121 10.1016/0966-842X(93)90112-5 PMC7133224

[5] Connolly SA, Leser GP, Jardetzky TS, Lamb RA. Bimolecular complementation of paramyxovirus fusion and hemagglutinin-neuraminidase proteins enhances fusion: implications for the mechanism of fusion triggering. Journal of virology. 2009;83:10857–10868. doi: 10.1128/JVI.01191-09 19710150 10.1128/JVI.01191-09 PMC2772755

[6] Peeters BP, de Leeuw OS, Koch G, Gielkens AL. Rescue of Newcastle disease virus from cloned cDNA: evidence that cleavability of the fusion protein is a major determinant for virulence. Journal of virology. 1999;73:5001–5009. 10233962 10.1128/jvi.73.6.5001-5009.1999 PMC112544

[7] Hernandez LD, Hoffman LR, Wolfsberg TG, White JM. Virus-cell and cell-cell fusion. Annu Rev Cell Dev Biol. 1996;12:627–661. doi: 10.1146/annurev.cellbio.12.1.627 8970739 10.1146/annurev.cellbio.12.1.627

[8] Kawahara N, Yang XZ, Sakaguchi T, Kiyotani K, Nagai Y, Yoshida T. Distribution and substrate specificity of intracellular proteolytic processing enzyme(s) for paramyxovirus fusion glycoproteins. The Journal of general virology. 1992;73:583–590. doi: 10.1099/0022-1317-73-3-583 1312118 10.1099/0022-1317-73-3-583

[9] Murakami M, Towatari T, Ohuchi M, Shiota M, Akao M, Okumura Y, et al Mini-plasmin found in the epithelial cells of bronchioles triggers infection by broad-spectrum influenza A viruses and Sendai virus. Eur J Biochem. 2001;268:2847–2855. 11358500 10.1046/j.1432-1327.2001.02166.x

[10] de Leeuw OS, Hartog L, Koch G, Peeters BP. Effect of fusion protein cleavage site mutations on virulence of Newcastle disease virus: non-virulent cleavage site mutants revert to virulence after one passage in chicken brain. The Journal of general virology. 2003;84:475–484. doi: 10.1099/vir.0.18714-0 12560582 10.1099/vir.0.18714-0

[11] Panda A, Huang Z, Elankumaran S, Rockemann DD, Samal SK. Role of fusion protein cleavage site in the virulence of Newcastle disease virus. Microbial Pathogenesis. 2004;36:1–10. doi: 10.1016/j.micpath.2003.07.003 14643634 10.1016/j.micpath.2003.07.003 PMC7125746

[12] Li Z, Sergel T, Razvi E, Morrison T. Effect of cleavage mutants on syncytium formation directed by the wild-type fusion protein of Newcastle disease virus. Journal of virology. 1998;72:3789–3795. 9557661 10.1128/jvi.72.5.3789-3795.1998 PMC109601

[13] Fujii Y, Kiyotani K, and Yoshida T. comparison of substrate specificities against the fusion glycoprotein of virulent NDV between a chick embryo fibroblast processiong protease and mammalian subtilisin-like proteases. Microbiol Immunol. 1999;43:133–140. 10229267 10.1111/j.1348-0421.1999.tb02384.x

[14] Xiao S, Nayak B, Samuel A, Paldurai A, Kanabagattebasavarajappa M, Prajitno TY, et al Generation by reverse genetics of an effective, stable, live-attenuated newcastle disease virus vaccine based on a currently circulating, highly virulent Indonesian strain. PLoS One. 2012;7:e52751 doi: 10.1371/journal.pone.0052751 23285174 10.1371/journal.pone.0052751 PMC3528709

[15] Samal S, Kumar S, Khattar SK, Samal SK. A single amino acid change, Q114R, in the cleavage-site sequence of Newcastle disease virus fusion protein attenuates viral replication and pathogenicity. The Journal of general virology. 2011;92:2333–2338. doi: 10.1099/vir.0.033399-0 21677091 10.1099/vir.0.033399-0

[16] Subbiah M, Khattar SK, Collins PL, Samal SK. Mutations in the fusion protein cleavage site of avian paramyxovirus serotype 2 increase cleavability and syncytium formation but do not increase viral virulence in chickens. Journal of virology. 2011;85:5394–5405. doi: 10.1128/JVI.02696-10 21450835 10.1128/JVI.02696-10 PMC3094964

[17] Diel DG, da Silva LH, Liu H, Wang Z, Miller PJ, Afonso CL. Genetic diversity of avian paramyxovirus type 1: proposal for a unified nomenclature and classification system of Newcastle disease virus genotypes. Infect Genet Evol. 2012;12:1770–1779. doi: 10.1016/j.meegid.2012.07.012 22892200 10.1016/j.meegid.2012.07.012

[18] Benson DA, Clark K, Karsch-Mizrachi I, Lipman DJ, Ostell J, Sayers EW. GenBank. Nucleic Acids Res. 2015;43:D30–D35. doi: 10.1093/nar/gku1216 25414350 10.1093/nar/gku1216 PMC4383990

[19] Schneider TD, Stephens RM. Sequence logos: a new way to display consensus sequences. Nucleic Acids Res. 1990;18:6097–6100. 2172928 10.1093/nar/18.20.6097 PMC332411

[20] Sergel T, Peeples ME, and Morrison TG. The attachment function of the Newcastle Disease Virus Hemagglutinin-Neuraminidase Protein can be speparated from fusion promotion by mutation. Virology. 1993;193:717–726. doi: 10.1006/viro.1993.1180 8384752 10.1006/viro.1993.1180

[21] Gravel KA, McGinnes LW, Reitter J, Morrison TG. The transmembrane domain sequence affects the structure and function of the Newcastle disease virus fusion protein. Journal of virology. 2011;85:3486–3497. doi: 10.1128/JVI.02308-10 21270151 10.1128/JVI.02308-10 PMC3067846

[22] Kattenbelt JA, Stevens MP, Gould AR. Sequence variation in the Newcastle disease virus genome. Virus research. 2006;116:168–184. doi: 10.1016/j.virusres.2005.10.001 16430984 10.1016/j.virusres.2005.10.001

[23] Tse LV, Hamilton AM, Friling T, Whittaker GR. A novel activation mechanism of avian influenza virus H9N2 by furin. Journal of virology. 2014;88:1673–1683. doi: 10.1128/JVI.02648-13 24257604 10.1128/JVI.02648-13 PMC3911587

[24] Collins MS, and Alexande DJ. Deduced amino acid sequences at the fusion protein cleavage site of Newcastle disease viruses showing variation in antigenicity and pathogenicity. Arch Virol. 1993;128:363–370. 8435046 10.1007/BF01309446

[25] Padhi A, Ma L. Time-dependent selection pressure on two arthropod-borne RNA viruses in the same serogroup. Infect Genet Evol. 2015;32:255–264. doi: 10.1016/j.meegid.2015.03.019 25801608 10.1016/j.meegid.2015.03.019

[26] Roebroek AJ, Creemers JW, Ayoubi TA, Van de Ven WJ. Furin-mediated proprotein processing activity: involvement of negatively charged amino acid residues in the substrate binding region. Biochimie. 1994;76:210–216. 7819325 10.1016/0300-9084(94)90148-1

[27] Kim SH, Chen Z, Yoshida A, Paldurai A, Xiao S, Samal SK. Evaluation of fusion protein cleavage site sequences of Newcastle disease virus in genotype matched vaccines. Plos One. 2017;12(3):e0173965 doi: 10.1371/journal.pone.0173965 28339499 10.1371/journal.pone.0173965 PMC5365116

